# A major isoform of mitochondrial *trans*-2-enoyl-CoA reductase is dispensable for wax ester production in *Euglena gracilis* under anaerobic conditions

**DOI:** 10.1371/journal.pone.0210755

**Published:** 2019-01-16

**Authors:** Takuya Tomiyama, Kyo Goto, Yuji Tanaka, Takanori Maruta, Takahisa Ogawa, Yoshihiro Sawa, Takuro Ito, Takahiro Ishikawa

**Affiliations:** 1 Department of Life Science and Biotechnology, Faculty of Life and Environmental Science, Shimane University, Matsue, Shimane, Japan; 2 Core Research for Evolutional Science and Technology (CREST), Japan Science and Technology Agency (JST), Chiyoda-ku, Tokyo, Japan; 3 Institute for Advanced Biosciences, Keio University, Tsuruoka, Yamagata, Japan; 4 Systems Biology Program, Graduate School of Media and Governance, Keio University, Fujisawa, Japan; University of Illinois, UNITED STATES

## Abstract

Under anaerobic conditions, *Euglena gracilis* produces a large amount of wax ester through mitochondrial fatty acid synthesis from storage polysaccharides termed paramylon, to generate ATP. *Trans*-2-enoyl-CoA reductases (TERs) in mitochondria have been considered to play a key role in this process, because the enzymes catalyze the reduction of short chain length CoA-substrates (such as crotonyl-CoA). A TER enzyme (EgTER1) has been previously identified and enzymologically characterized; however, its physiological significance remained to be evaluated by genetic analysis. We herein generated EgTER1-knockdown *Euglena* cells, in which total crotonyl-CoA reductase activity was decreased to 10% of control value. Notably, the knockdown cells showed a severe bleaching phenotype with deficiencies in chlorophylls and glycolipids, but grew normally under heterotrophic conditions (with glucose supplementation). Moreover, the knockdown cells accumulated much greater quantities of wax ester than control cells before and after transfer to anaerobic conditions, which was accompanied by a large metabolomic change. Furthermore, we failed to find any contribution of other potential *TER* genes in wax ester production. Our findings propose a novel role of EgTER1 in the greening process and demonstrate that this enzyme is dispensable for wax ester production under anaerobic conditions.

## Introduction

*Euglena gracilis* is a single-celled eukaryotic alga containing chloroplasts with photosynthetic activity. Photosynthetic products are stored as polysaccharides termed paramylon, which consist of only liner β-1,3-glucan, in *Euglena* grown under aerobic conditions [1,2]. Notably, paramylon content often exceeds 50% of the dry weight of the cell [3,4]. Recently, one of two glucan synthase-like proteins, EgGSL2, has been found to be required for paramylon synthesis [5]. When *Euglena* cells are placed in anaerobiosis, paramylon is rapidly degraded and used for the synthesis of wax esters. Wax esters produced by *Euglena* consist of saturated fatty acids and alcohols with carbon chain-lengths of 10 through 18 including odd-numbered chain lengths, with the major constituents being myristic acid (C14:0) and myristyl alcohol (C14:0) [6]. The process of converting paramylon to wax esters involves glycolysis that produces ATP from substrate-level phosphorylation; therefore, this phenomenon has been termed “wax ester fermentation” [7,8].

At least three pathways for fatty acid synthesis have been proposed in *Euglena*: 1) *via* a multifunctional fatty acid synthase (FAS I) in the cytosol, 2) an acyl carrier protein-dependent system (FAS II) in illuminated chloroplasts, and 3) malonyl-CoA-independent fatty acid synthesis in mitochondria. The cytosolic and chloroplastic pathways require malonyl-CoA, which is synthesized in an ATP-dependent manner [9–11]. In contrast, the mitochondrial pathway does not require malonyl-CoA and can synthesize fatty acids with acetyl-CoA as both primer and C_2_ donor, using NAD(P)H as an electron donor [12]. Thus, the mitochondrial system operates in an ATP-independent manner, allowing the net gain of ATP during the wax ester fermentation process.

**Fig 1:**
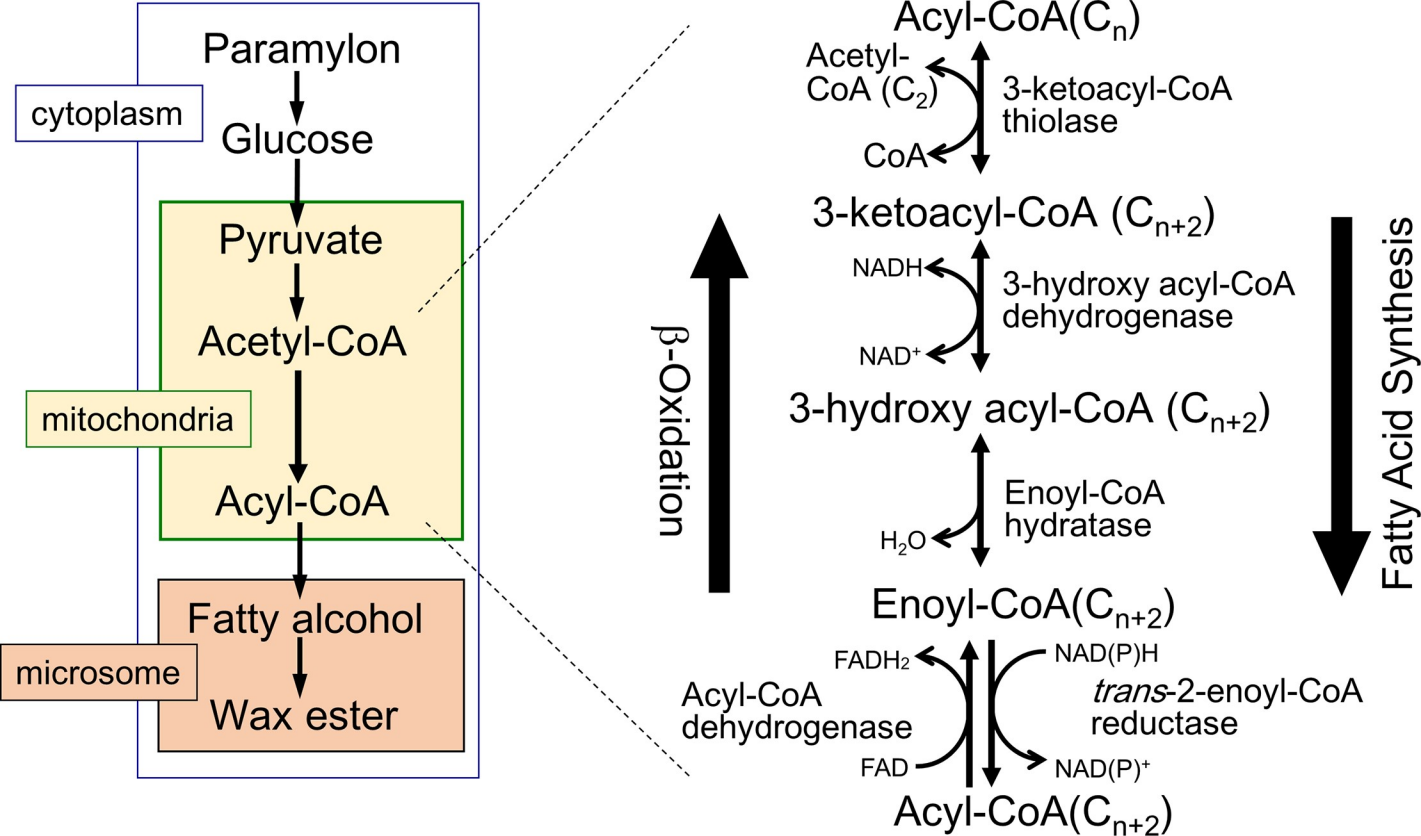
Overview of the pathway for wax ester synthesis in *Euglena gracilis*. Following glycolysis in the cytosol, fatty acids are synthesized by the reverse reaction of β-oxidation as highlighted in the right side of the figure. *trans*-Enoyl-CoA reductase plays a key role in the fatty acid synthesis, rather than acyl-CoA dehydrogenase.

Generally, the fatty acid synthetic pathway in mitochondria is the reverse of the ß-oxidation pathway, with one exception being that *trans*-2-enoyl-CoA reductase (TER) is used instead of acyl-CoA dehydrogenase, which is considered to catalyze irreversible reaction in the β-oxidation pathway (Fig 1). Instead, the *Euglena* TER can preferably catalyze the reduction of short chain length substrates (such as crotonyl-CoA). This feature allows the mitochondrial system to act as a *de novo* fatty acid synthetic pathway. Among the TER isoforms, one was previously purified from *Euglena* mitochondria and its cDNA sequence was determined [13]. This enzyme, herein termed EgTER1 (see below), exhibited a preferential activity toward crotonyl-CoA using both NADH and NADPH as electron donors [13]. TER activity in *Euglena* was found to be extremely low compared to that of the other enzymes in the mitochondrial fatty acid synthesis pathway (i.e., 3-ketoacyl-CoA thiolase, 3-hydroxyacyl-CoA dehydrogenase and *trans*-2-enoyl-CoA hydratase) [12–14]. In addition, TER activity was expected to be important for NAD^+^ supply for the glycolytic pathway. For these reasons, TER was considered as the rate-limiting step in wax ester fermentation. However, this scenario has been proposed only based on biochemical studies without any molecular cell biological evidence.

Accordingly, the aim of this study was to investigate the physiological significance of TER enzyme(s) in wax ester fermentation using a reverse genetic approach. Herein we succeeded in generating *Euglena* cells exhibiting knock down of the *EgTER1* gene but, unexpectedly, failed to find any contribution of the enzyme to wax ester production under anaerobic conditions. Rather, we found this enzyme to be involved in greening process (chlorophyll synthesis and/or chloroplast development).

## Materials and methods

### Strain and culture

*E*. *gracilis* strain Z was grown in Koren-Hutner (KH) medium for heterotrophic growth [15] or Cramer-Myers (CM) medium for autotrophic growth [16] under continuous light conditions (50 μmol m^-2^ s^-1^) at 26°C for 6–14 days, by which time the stationary phase was reached. *E*. *gracilis* SM-ZK, a non-photosynthetic mutant strain [17], was cultured in KH medium with aeration at 26 ˚C for 6 days to the late logarithmic phase of growth. Aerobic cultures were grown with continuous shaking (120 rpm) under atmospheric conditions, and the anaerobic cultures were completely sealed and allowed to stand for 24 h after the replacement of air by N_2_ gas. Cell number and volume were determined using an electric field multi-channel cell counting system, CASY (Roche Diagnostics, Basel, Switzerland).

### Gene knockdown experiments

Knockdown of *TER* genes was performed as described previously [5,18]. Template cDNAs were polymerase chain reaction (PCR)-amplified using primers including the T7 RNA polymerase promoter sequence at one end as listed in S1 Table. Then, the dsRNAs were synthesized from the template cDNAs using the MEGAscript RNAi Kit (Thermo Fisher Scientific, MA) by following the manufacturer’s instructions. *Euglena* cells of 2-d-old cultures were collected and resuspended in culture medium containing 4.2 mM Ca(NO_3_)_2_, 3.7 mM KH_2_PO_4_, and 2.1 mM MgSO_4_. The cell suspension (100 μL; approximately 5 × 10^6^ cells) was transferred into a 0.4-cm-gap cuvette and electroporated with 5 μL of RNA solution (15 μg of dsRNA in 50 mM Tris-HCl, pH 7.5, and 1 mM ethylenediaminetetraacetic acid) using the NEPA21 electroporator (Nepa Gene, Chiba, Japan). The parameters were as follows: voltage 250V; pulse length, 3 ms; pulse interval, 50 ms; number of pulses, 2; decay rate; 10%; polarity + as poring pulse and voltage, 20 V; pulse length, 50 ms; pulse interval, 50 ms; number of pulses, 5; decay rate; 40%; polarity +/- as transfer pulse. Subsequently, the cell suspension was diluted with fresh liquid KH medium and cultured at 26°C for 7 days for restoration.

### Reverse transcription (RT)-PCR

Total RNA was prepared from wild-type and dsRNA-introduced *Euglena* cells using the RNAiso regent (TaKaRa, Shiga, Japan). The first strand cDNA was synthesized using a PrimeScript II 1^st^ strand cDNA kit (TaKaRa) with an oligo(dT) primer. The PCR was performed using specific primers as listed in S1 Table. EF1-α from *Euglena* (X16890) was used as a normalization gene.

### Recombinant TER preparation

The open reading frames of *Euglena* TER were amplified from the first strand cDNA pool using the primer sets as listed in S1 Table. The amplified DNA fragments were ligated into the pGEM-T easy vector (Promega, Madison, WI, USA) to confirm the absence of PCR errors. The resulting constructs were digested with suitable restriction enzymes and were ligated into the expression vector pCold II (TaKaRa) to produce His-tagged proteins. The resulting plasmids were introduced into *Escherichia coli* strain BL21 Star cells (Agilent Technologies, Santa Clara, CA, USA). The *E*. *coli* cells transformed with each construct were grown in 3 mL of Luria-Bertani (LB) medium containing 50 μg mL^-1^ ampicillin. After an overnight culture at 37°C, the cultures were transferred to 600 mL of LB medium (with ampicillin) and grown to an A600 of 0.5. Isopropyl-1-thio-β-D-galactopyranoside was added to a concentration of 0.5 mM and the cells were incubated for 20 h at 15°C. The harvested cells were resuspended in 100 mM potassium phosphate buffer, pH 7.0 and sonicated. His-tagged recombinant EgTER proteins were purified using a TALON Metal Affinity resin (Clontech, Mountain View, CA, USA) according to the manufacturer’s instructions. Protein contents were determined using Bradford reagents. The purified enzymes were then desalted and concentrated using an ultrafiltration membrane (Amicon Ultra-4, Millipore, Billerica, MA, USA), and stored at −20°C until use.

### TER activity measurement

TER activity was measured as described previously [12]. The reaction mixture contained 100 mM potassium phosphate buffer, pH 6.2, 0.4 mM NADPH, 0.5 mM crotonyl-CoA, and enzyme. The oxidation of NADPH was followed by a decrease in absorbance at 340 nm (ε = 6.22 mM^-1^ cm^-1^). The protein concentration of enzyme solution was measured using the Coomassie Blue protein assay reagent (Bio-Rad Laboratories, Hercules, CA, USA).

### Determination of chlorophyll

For extraction of chlorophyll, *Euglena* cells harvested from 1 mL of culture were vortexed in 1 mL of 80% (v/v) acetone. After centrifugation at 10,000 *g* for 5 min, chlorophyll in the supernatant was measured as described by Arnon [19].

### Wax ester measurement

Extraction of the total wax ester fraction from *Euglena* cells was performed according to the method described by Inui et al [7]. *Euglena* cells were harvested, freeze-dried, and added to 2.4 mL of a mixture of chloroform, methanol, and water in the ratio 10:20:8 (v/v/v). After thorough agitation, the mixture was centrifuged to remove cells and debris. The extraction was repeated, and the combined supernatants were added to 1 mL each of chloroform and water, followed by vigorous shaking. After centrifugation, the chloroform phase was evaporated and dissolved in hexane. The wax ester fraction was filtered using a polytetrafluoroethylene 0.22-μm filter for gas chromatography-mass spectrometry (GC-MS) analysis. The wax ester fraction was separated and determined using a GCMS-QP2010 instrument (Shimadzu, Kyoto, Japan). Separation was performed on an Agilent J&W DB-5ms column (30 m × 0.25 mm internal diameter, 0.25 μm film thickness; Agilent Technologies). A 1-μL portion of the wax ester fraction was injected into GC-MS using a splitless injection; helium was used as the carrier gas (1.16 mL/min). Chromatographic separation was initially set at 100°C (1 min), then the temperature was increased to 280°C (10°C per min) and held for 10 min. The mass transfer line and ion source were at 250°C. Myristyl myristate was detected using electron ionization (70 eV) in the selected ion monitoring mode at m/z 229.2 and 57.1 for the quantitative analysis.

### Fatty acids measurement

*Euglena* cells were harvested, freeze-dried, and then the whole fatty acids were methyl esterified using a Fatty Acid Methylation kit and FAME purification kit (Nacalai Tesque, Kyoto, Japan). The purified FAME was analyzed by GC-MS with the condition described above by using the FAME compound library supplied by the manufacture.

### Paramylon measurement

Paramylon contents were determined using the phenol-sulfuric acid method with glucose solution as a standard as previously described by Tanaka et al [5].

### Metabolome analyses

Approximately 10^7^ cells were collected from three individual cultures of aerobic and anaerobic-treated *Euglena* cells for analysis. Anaerobic treatment was performed as follows: a 5 mL of aliquot of the aerobic cell culture was dispensed into 15 mL plastic tube and bubbled with N_2_ gas for 1 min. Tube were then tightly capped and allowed to stand by shading for 24 h. The metabolite was extracted by chloroform–methanol–water at 1:1:0.4 (v/v) as previously described [20]. The capillary electrophoresis–time-of-flight mass spectrometry (CE–TOFMS) and liquid chromatography (LC)–TOFMS conditions for cationic and anionic metabolite, and lipid analyses were as described elsewhere [20–23]. Raw data from the TOFMS analyses were processed using MasterHands software [21–23]. For the CE–TOFMS analysis, all the compounds, including cationic and anionic compounds, were identified by matching them to standard compounds in the Keio University’s library. For the LC–TOFMS analysis, several major compounds were also identified in this manner, with the others identified based on theoretical m/z values with mass accuracy of 20 ppm and orderly shift of retention time, described by Ikeda et al [24]. Quantities were calculated using an internal standard: methionine sulfone for cationic metabolites, D-camphor-10-sulfonic acid for anionic metabolites, and dihexanoyl (d22)-*sn*-glycero-3-phosphocholine for lipids analyses. The position of double bonds or chirality was not identified in this study. All peaks were visually confirmed.

### Data analysis

The significant of differences between data sets were evaluated using Student’s *t*-tests. Calculations were carried out using Microsoft Excel software.

## Results

### Generation and characterization of EgTER1 knockdown cells

**Fig 2:**
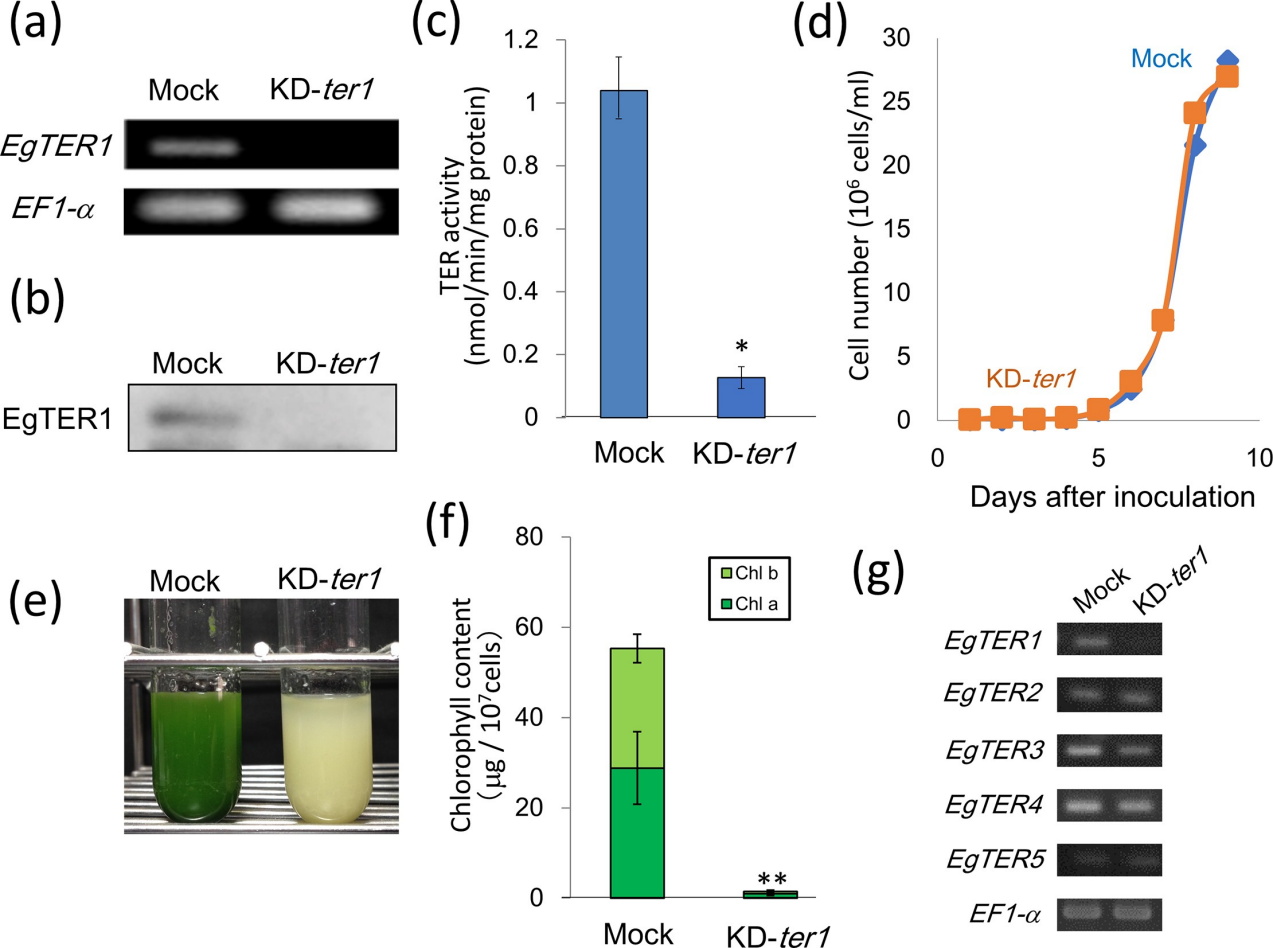
Effect of *EgTER1*-knockdown on cell growth and chlororphyll content in heterotrophically grown cells. (a) RT-PCR for verification of *EgTER1* gene knockdown. RT-PCR was carried out using total RNA from *Euglena* cells in which dsRNA was introduced. Mock cells were electroporated without dsRNA. (b) Immuno detection of TER1 protein. (c) Comparison of TER activity between mock control and KD-*ter1* cells. *Euglena* cells grown to stationary phase were collected for TER activity measurement as described in the Material and Method section. An asterisk denotes statistically significant differences (*P<0.05) compared with the mock control. Values represent the means ± SD of three independent experiments. (d) Growth curve of mock control (blue) and KD-*ter1* (orange) cells. The cultures were incubated in the heterotrophic KH medium. (e) Green color-less phenotype of KD-*ter1* cells. The picture shows a representative culture after 7 days growth in the KH medium. (f) Influence of *EgTER1*-knockdown on chlorophyll content. An asterisk denotes statistically significant differences (**P< 0.01) compared with the mock control. Values represent the means ± SD of three independent experiments. (g) Effect of gene expression levels of other TER orthologs on *EgTER1*-knockdown. RT-PCR was carried out using total RNA from *Euglena* cells in which dsRNA was introduced.

To study the physiological significance of EgTER1, its knockdown cells (KD-*ter1*) were generated through electroporation-mediated dsRNA introduction based on established procedures [5,18]. Notably, when grown under heterotrophic conditions, the dsRNA-introduced cells exhibited a severe bleaching phenotype with a deficiency in chlorophyll (Fig 2E and 2F). Semi-quantitative RT-PCR and protein blotting confirmed that EgTER1 expression was markedly reduced in the knockdown cells compared to that in the mock control (Fig 2A and 2B). This resulted in an approximately 90% reduction in total TER activity against crotonyl-CoA (Fig 2C), indicating that EgTER1 constitutes a major isoform that recognizes a short chain CoA like crotonyl-CoA.

Despite its bleaching phenotype, growth of KD-*ter1* was indistinguishable from that of mock control cells under the heterotrophic conditions (Fig 2D). Thus, the colorless phenotype was not due to any apparent cellular damage caused by EgTER1 knockdown. Conversely, when grown under autotrophic conditions (without sugar supplementation), an obvious growth retardation of KD-*ter1* cells was observed. However, its bleaching phenotype was considerably restored under these conditions (S1 Fig). This might have occurred because cells with a low RNAi effect may have preferentially grown under the conditions provided following dsRNA introduction.

To address whether the bleaching phenotype was due to knockdown of the *EgTER1* gene in more detail, knockdown experiments were carried out using other regions of the *EgTER1* sequence as triggers for RNAi (S2 Fig). In all cases, greening was impaired in KD-*ter1* cells with reduced transcript levels of *EgTER1*. These results indicated that EgTER1 is required for the greening process in *Euglena*. It should be noted that the *EgTER1* knockdown did not affect the expression of other potential *TER* genes (i.e., *EgTER2-5*, see the last subsection of ‘Results‘) indicated from our previous RNA-Seq analysis [25] (Fig 2G).

### Wax ester fermentation in EgTER1 knockdown cells

**Fig 3:**
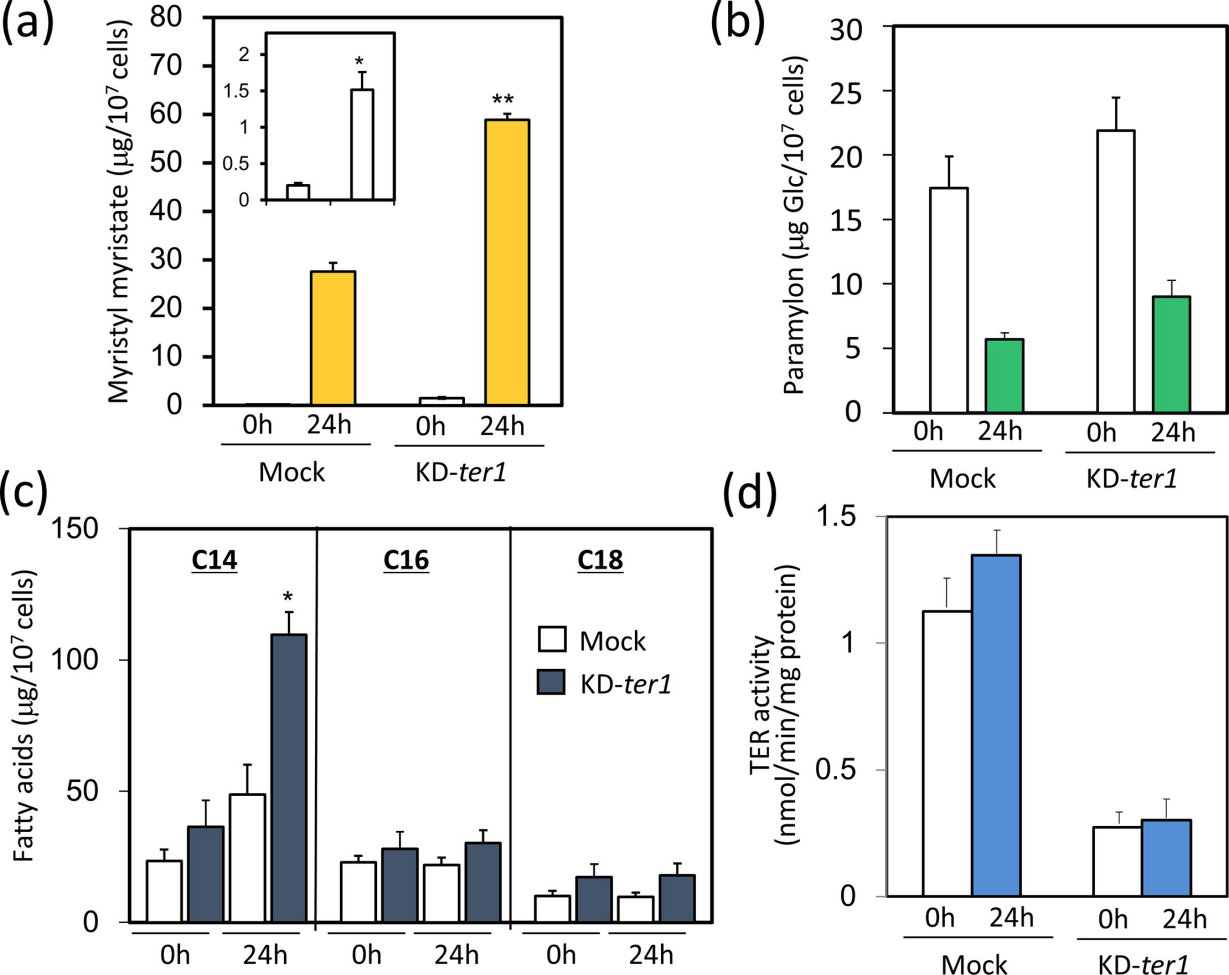
Effect of *EgTER1*-knockdown on some key compounds in wax ester fermentaion. (a) Influence of *EgTER1*-knockdown on myristyl myristate production under anaerobic conditions. *Euglena* cells grown to stationary phase were anaerobically treated for 24 h and then collected for wax ester measurement as described in the Material and Method section. The inset shows the results for 0 h in detail. An asterisk denotes statistically significant differences (*P<0.05, **P< 0.01) compared with the mock control. Values represent the means ± SD of three independent experiments. (b) Paramylon contents in *EgTER1*-knockdown cells. Cells grown to stationary phase were anaerobically treated for 24 h and collected for paramylon measurement. Values represents the means ± SD of three independent experiments. (c) Fatty acid content. Cells grown to stationary phase were anaerobically treated for 24 h and collected for fatty acids measurement. Values represent the means ± SD of three independent experiments. An asterisk denotes statistically significant differences (*P<0.05) compared with the mock control. (d) TER activity prior to and following anaerobic treatment. Values represent the means ± SD of three independent experiments.

Next, wax ester production in KD-*ter1* cells was evaluated. Considering its impact on cell growth, the following analyses were performed using *Euglena* cells grown under heterotrophic conditions unless otherwise stated. Control and KD-*ter1* cells grown under heterotrophic conditions with air were transferred to anaerobic conditions. In response to this treatment, levels of myristyl myristate (C_28_), a major component of *Euglena* wax esters, were markedly increased in both control and knockdown cells (Fig 3A). However, the accumulation was slightly albeit more pronounced in KD-*ter1* cells compared to that in the control. Moreover, even prior to anaerobic treatment, myristyl myristate accumulation was higher in KD-*ter1* cells. A comparable result was observed when EgTER1 was knocked down using different RNAi trigger regions (S2 Fig). Levels of methyl myristate (C_14_), a precursor of C_28_ wax ester, were also significantly higher in KD-*ter1* cells, although knockdown of EgTER1 did not affect other fatty acids (C_16_ and C_18_) (Fig 3C). A fatty acid profile analysis also indicated that the amount of fatty acids with the carbon length ranging from C12 to C15 increased equally, clearly supporting the results of the wax ester accumulation in TER1 silencing cells (S3 Fig). These observations were unlikely to result from compensation by other potential TER enzyme(s), because KD-*ter1* cells retained a low activity toward crotonyl-CoA even during anaerobic stress (Fig 3D). Conversely, paramylon accumulation and degradation were not affected by the EgTER1 knockdown under aerobic conditions and subsequent anaerobic treatment (Fig 3B).

Because KD-*ter1* cells were severely bleached, the marked wax accumulation may have occurred because of a secondary effect of the loss of functional chloroplasts. To address this issue, the same knockdown assay was performed on a *Euglena* strain lacking chloroplasts (SM-ZK). As a result, the accumulation of myristyl myristate was again more pronounced in the KD-*ter1* cells than that in the control under anaerobic conditions. Thus, our findings indicated that *Euglena* could produce wax esters without EgTER1 activity (i.e., the crotonyl-CoA reduction activity).

### Metabolomic profile of TER1 knockdown cells

**Fig 4:**
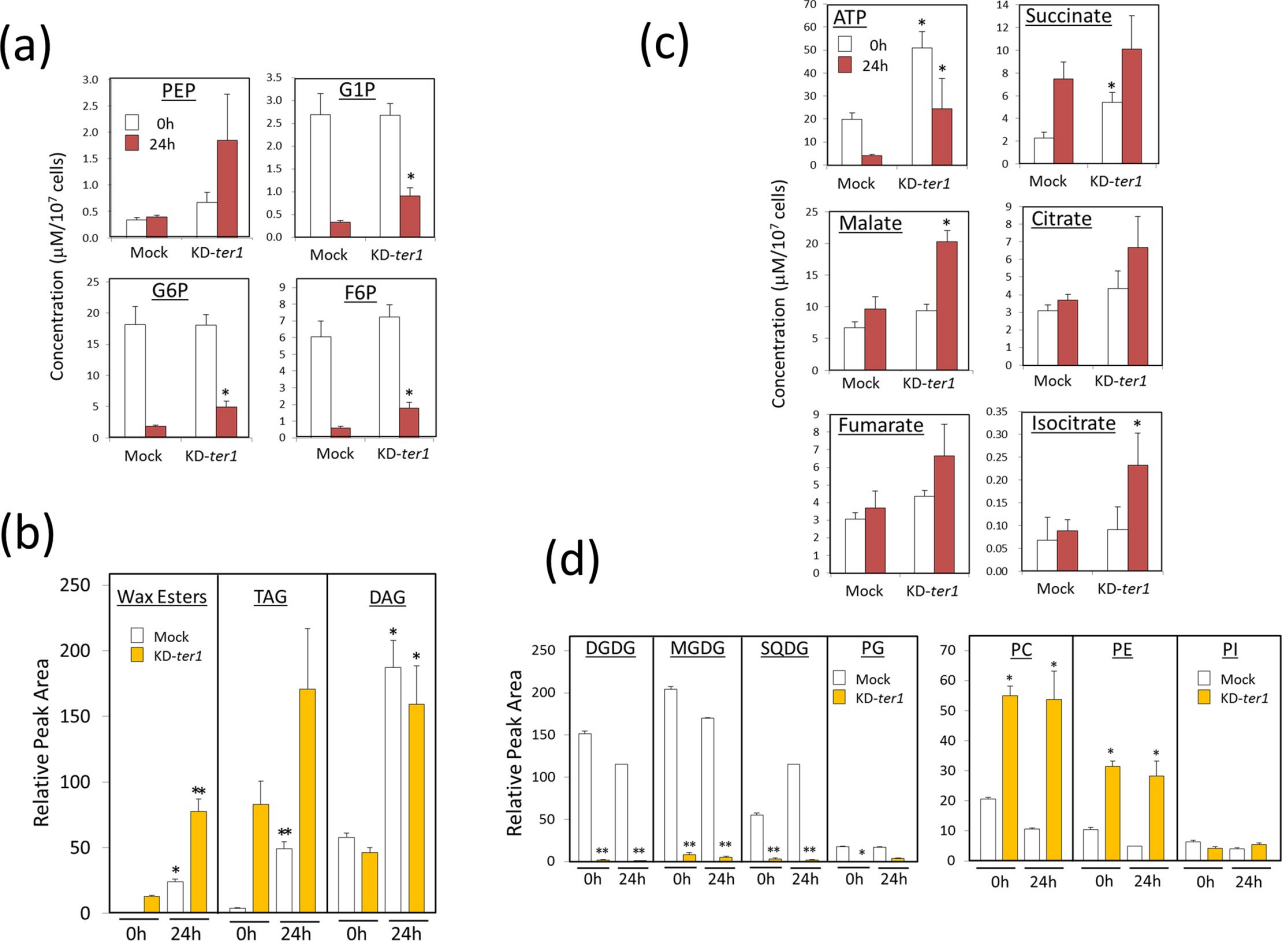
**Concentrations of some key metabolites related to the glycolysis (a), TCA cycle (c), storage lipid (c) and membrane lipid (d) metabolism.** Cells grown to stationary phase were anaerobically treated for 24 h and collected for metabolome analysis. Concentrations and relative amounts of each metabolite in the mock control and *EgTER1*-knockdown cells are shown. An asterisk denotes statistically significant differences (*P<0.05, **P< 0.01) compared with the mock control. Values represent the means ± SD of three independent experiments.

Metabolome analysis was next performed using KD-*ter1* cells prior to (0 h) and following (24 h) anaerobic treatment to determine the further implications for the effect of EgTER1 on metabolism. This encompassed some kinds of lipids, sugars, amino acids, and ATP. The enhanced production of wax ester in KD-*ter1* cells was also observed in this metabolome analysis (Fig 4). However, glycolipids, such as monogalactosyl diacylglycerol (MGDG), digalactosyl diacylglycerol (DGDG), sulfoquinovosyldiacylglycerol (SQDG), and phosphatidylglycerol (PG), all of which are components of chloroplast membranes, were poorly detected in KD-*ter1* cells. This was consistent with the colorless phenotype of the knockdown cells. Levels of phosphatidylcholine (PC) and phosphatidylethanolamine (PE), being glycerophospholipids, were higher in the knockdown cells compared to the control both before and after anaerobic treatment. As the PC is a precursor of chloroplastic glycolipids, MGDG, DGDG and SQDG, it is reasonable to conclude that the higher accumulation of both PC and PE occurs as a result of the suppression of these biosynthesis in the knockdown cells. In the control cells, ATP levels were markedly decreased upon anaerobic treatment, which was also the case in KD-*ter1* cells. Conversaly, ATP levels in KD-*ter1* cells were much higher than those in the control cells both prior to and following anaerobic treatment. As shown in Table 1, the knockdown of *EgTER1* had a large effect on the levels of amino acids. For example, in KD-*ter1* cells, the levels of alanine, glycine, tryptophan, and arginine were highly accumulated under aerobic conditions, then significantly decreased upon anaerobic treatment. Thus, the *EgTER1* knockdown perturbed several metabolic pathways, which might result in the notable accumulation of wax ester.

**Table 1:** Concentrations of amino acids in *TER1*-knocked down cells after anaerobic treatment for 24 h.

	Concentration (μM/10^6^ cells)			
	Mock 0h	Mock 24h	KD-*ter1* 0h	KD-*ter1* 24h
Ala	3.98 ± 0.82	4.43 ± 0.99	49.1 ± 26.1	28.7* ± 4.31
Gly	2.45 ± 0.42	1.29 ± 0.05	9.00 ± 3.10	3.79* ± 0.29
Ser	1.11 ± 0.14	0.72 ± 0.06	1.63 ± 0.14	1.48 ± 0.46
Thr	1.03 ± 0.11	1.76 ± 0.07	2.11 ± 0.55	3.04 ± 0.79
Trp	0.20 ± 0.02	0.55 ± 0.03	7.03 ± 3.27	2.36* ± 0.74
Ile	0.88 ± 0.12	1.21 ± 0.05	1.61* ± 0.39	2.46* ± 0.41
Met	0.18 ± 0.04	1.51 ± 0.16	0.43* ± 0.05	1.55 ± 0.28
Val	6.18 ± 1.00	5.51 ± 0.82	6.92 ± 1.44	6.13* ± 0.99
Asp	5.41 ± 0.77	2.20 ± 0.12	9.47* ± 2.28	15.2 ± 9.79
Asn	1.14 ± 0.23	0.68 ± 0.03	3.59* ± 0.96	2.34 ± 1.41
Arg	1.47 ± 0.29	1.93 ± 0.11	18.77 ± 9.61	6.35 ± 2.98
Glu	14.1 ± 1.66	7.04 ± 0.77	12.3 ± 2.48	15.6 ± 8.88
Gln	1.38 ± 0.18	1.94 ± 0.15	10.6 ± 5.21	6.99 ± 4.54
His	1.57 ± 0.30	2.56 ± 0.25	3.45 ± 1.82	12.6* ± 4.23
Pro	0.94 ± 0.14	1.79 ± 0.06	4.15 ± 1.72	10.6* ± 2.42
Phe	0.26 ± 0.04	1.13 ± 0.01	0.99* ± 0.33	1.27 ± 0.17
Tyr	0.47 ± 0.05	1.62 ± 0.08	2.72 ± 1.29	1.70 ± 0.23
Leu	0.95 ± 0.16	4.42 ± 0.29	1.97 ± 0.66	5.72* ± 0.69
Lys	14.40 ± 3.17	24.4 ± 0.71	26.1* ± 7.54	69.8 ± 19.8

Concentrations of each amino acid in the mock control and KD-*ter1* cells before anaerobic treatment (0 h) and after anaerobic treatment (24 h) are shown. Data represent the means ± SD of three independent experiments. Asterisks denote statistically significant differences (*P < 0.05) compared with the corresponding mock control.

### Other potential *TER* genes

Our recent RNA-Seq data [25] showed that there are five genes annotated as TER; comp34527_c0_seq1_5 (EgTER1), comp27719_c0_seq1_4 (EgTER2), comp22878_c0_seq1_1 (EgTER3), comp27434_c0_seq1_3 (EgTER4), and comp32876_c0_seq1_1 (EgTER5). The sequence of EgTER1 amino acids is identical to that of the TER enzyme identified by Hoffmeister et al [13]. Amino acid sequences of these enzymes show identity (ranging from 6.8 to 29.1%) and similarity (from 45.3 to 73.8%) to each other. A domain search using the Pfam database (http://pfam.xfam.org/, [26]) showed that only EgTER1 has an obvious Enoyl-CoA_reductase domain (PF12241), which is located between the NAD(P)H- and FAD-domains (S4 Fig). This analysis found some domains related to alcohol dehydrogenase in EgTER4 and 5, but failed to find any domain in EgTER2 and 3 (S4 Fig). Although EgTER1 has an N-terminal extension that contains two potential transmembrane domains (S4 Fig), which is a characteristic of *Euglena* plastidic proteins [27], this enzyme was purified from *Euglena* mitochondria, demonstrating its mitochondrial localization. Although the prediction of *Euglena* protein localization using protein localization prediction tools is generally difficult, two such tools, Target P and WoLF PSORT, predicted that EgTER2, EgTER3, and EgTER5 are mitochondrial, whereas EgTER4 is cytosolic (S2 Table).

To investigate whether these potential TERs could catalyze the reduction of short chain length substrates, such as crotonyl-CoA, we attempted to generate their recombinant proteins. All EgTER proteins, except for EgTER3, could be expressed in and purified from *E*. *coli* cells. Consistent with previous report [13], EgTER1 exhibited substantial crotonyl-CoA reductase activity (3.20 ± 0.35 μmol/min/mg protein). In contrast, other enzymes did not show any remarkable activity with crotonyl-CoA as a substrate, although we did not check whether these enzymes could reduce other potential substrates.

**Fig 5:**
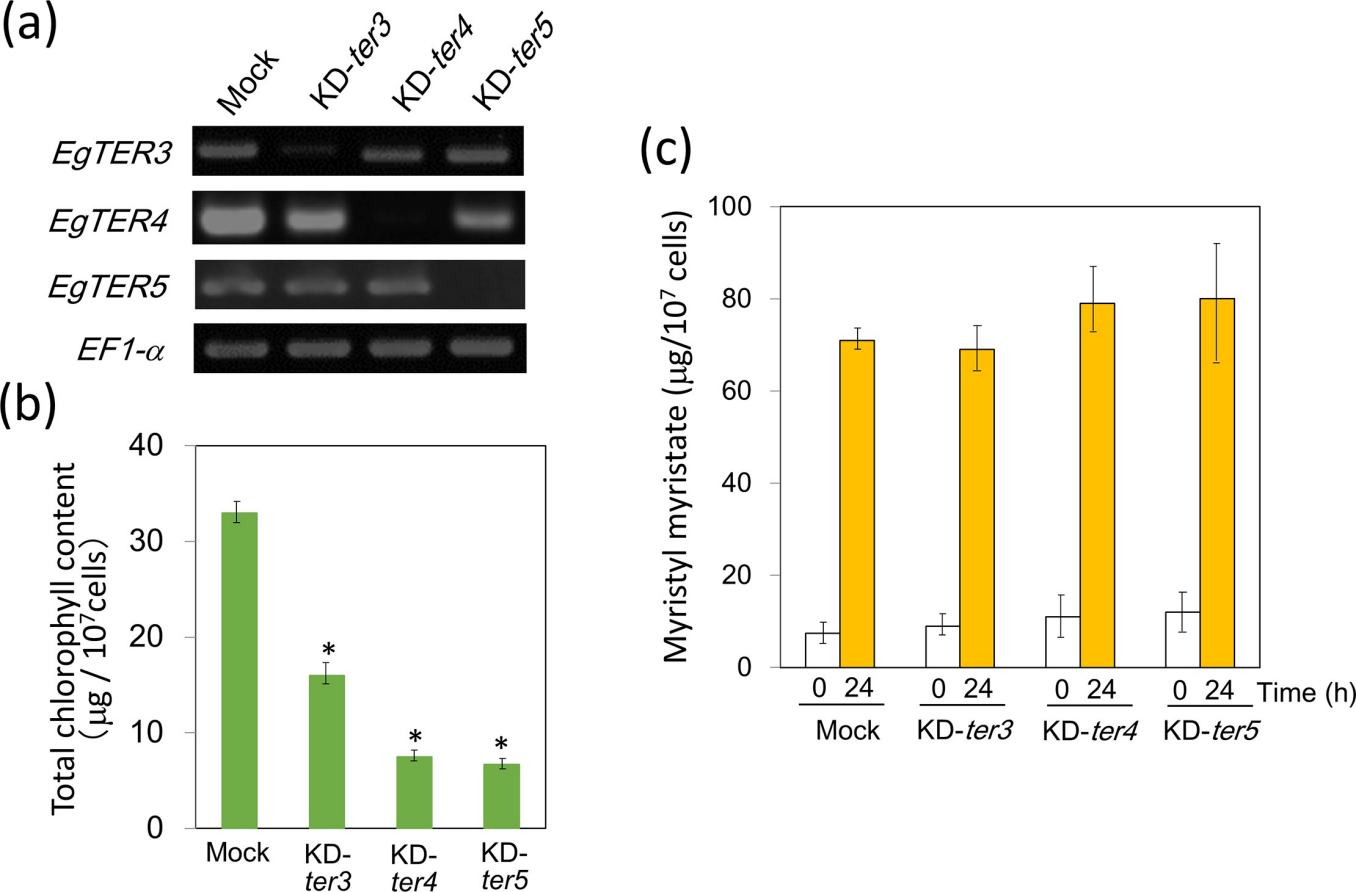
Effect of gene knockdown for other *TER* orthologs on wax ester production. (a) RT-PCR for verification of each possible *TER* gene knockdown. RT-PCR was carried out using total RNA from *Euglena* cells in which dsRNA was introduced. (b) Chlorophyll content in possible *TER* gene knockdown cells. An asterisk denotes statistically significant differences (*P<0.05) compared with the mock control. Values represent the means ± SD of three independent experiments. (c) Wax ester production under anaerobic conditions. *Euglena* cells grown to stationary phase were anaerobically treated for 24 h and collected for myristyl myristate measurement as described in the Material and Method section. Values represent the means ± SD of three independent experiments.

We also tested the effects of knockdown of these genes on wax ester production. KD-*ter2* cells could not grow even under heterotrophic conditions. In comparison, other knockdown cells (KD-*ter3*, *4* and *5*) grew normally but, as with KD-*ter1*, they accumulated less chlorophyll compared to the mock control under heterotrophic conditions (Fig 5B). Knockdown of these genes had no negative impact on wax ester production under anaerobic conditions (Fig 5C). These findings suggested that these enzymes do not act as crotonyl-CoA reductases in the wax ester fermentation process.

## Discussion

An outline of wax ester fermentation in *Euglena* upon anaerobic treatment was proposed by Inui et al [7]. At that time, it was still unclear how this process leads to the net gain of ATP, because only fatty acid synthesis pathways that require malonyl-CoA were known to operate in *Euglena*. That is, if these ATP-dependent pathways were involved in the wax ester production, the consumption of ATP was expected to exceed its generation in glycolysis. Soon thereafter, observation of TER activity against short chain length substrates (such as crotonyl-CoA) in mitochondria led to the proposal of a novel mitochondrial pathway for fatty acid synthesis that does not require ATP consumption, providing an explanation for the net gain of ATP in wax ester fermentation [8]. As previously noted, TER was strongly expected to constitute the rate-limiting enzyme in the mitochondrial pathway. The purpose of the present study was therefore to provide further evidence for the physiological role of TER in the fermentation process: however, no positive answer was provided from our reverse genetic analysis.

EgTER1 is identical to the enzyme cloned by Hoffmeister et al [13]. In the present study, approximately 90% reduction in total crotonyl-CoA reductase activity was found in KD-*ter1* cells, demonstrating the enzyme to be a major isoform. Nevertheless, knockdown of EgTER1 had no negative impact on wax ester production under anaerobic conditions. On the contrary, wax ester production was more pronounced in the knockdown cells as compared to that in the mock control. These findings demonstrated that EgTER1 is dispensable for wax ester production in *E*. *gracilis* under anaerobic conditions. However, the mechanism by which KD-*ter1* cells produced wax esters remains unknown. Judging from the primary structures and enzymological properties, other potential *EgTER* genes were unlikely to encode a crotonyl-CoA reductase. In comparison, Inui et al [28] purified an NADH-specific crotonyl-CoA reductase from *Euglena* mitochondria, although this has not yet been cloned. This isoform consisted of 15 and 25 kDa subunits and differed substantively from EgTER1, which has a molecular mass of 44 kDa. This enzyme might therefore be a component of the residual (approximately 10%) crotonyl-CoA reductase activity in KD-*ter1* cells. However, it should be noted that TER activity in *Euglena* is extremely low compared to the activity of the other enzymes in the mitochondrial fatty acid synthesis pathway [8,12–14]. Accordingly, the residual 10% crotonyl-CoA reductase activity that was retained in KD-*ter1* cells was unlikely to be sufficient to produce wax esters. Moreover, the residual activity cannot explain the pronounced wax ester synthesis in the knockdown cells. Alternative pathway(s) must therefore exist to substitute for the wax ester production in the absence of EgTER1. As shown by our metabolome analysis, large metabolic perturbations occurred in KD-*ter1* cells, which might result in the formation of new metabolic route(s) for fatty acid synthesis that might not be operating in the wild-type organism. Notably, genes involved in fatty acid biosynthesis were found to be upregulated in response to anaerobic conditions in our previous RNA-Seq analysis [25]. The increase in ketogenic amino acids like Leu, Ile, and Trp may be attributed to this phenomenon. Together, these observations suggest the existence of an alternative route for anaerobic wax ester synthesis. Recently, Nakazawa et al., [29] have reported that fatty acids are anaerobically synthesized in *Euglena* mitochondria by the reversal of β-oxidation, where *trans*-2-enoyl-CoA is reduced by acyl-CoA dehydrogenase (ACD1) using the electrons provided by NADH *via* the electron transport chain complex I, rhodoquinone, and electron transfer flavoprotein. Thus the reversal reaction of ACD1 would be one of the strong alternative possibilities. However, the contribution of ACD1 still admits of further argument to reach a crucial conclusion, because the ACD1 gene silencing cells show approximately 60% of residual accumulation of wax esters [29].

Another notable finding was that KD-*ter1* showed a bleaching phenotype concomitant with negligible levels of chlorophylls and glycolipids, indicating a critical role of EgTER1 for greening process (chloroplast development and/or chlorophyll biosynthesis). However, the role of this enzyme in greening is currently unknown. It is unlikely that the chlorophyll-less phenotype was due to cellular damage caused by EgTER1 silencing, because the knockdown cells grew similar to the mock control cells level under heterotrophic conditions. Alternatively, EgTER1 contains two transmembrane helices in its N-terminal region, which is a typical property of chloroplast-targeted proteins in *Euglena* [27]. Together with this characteristic, the high similarity between EgTER1 and FabV (that is enoyl-acyl-ACP reductase) sequences allows the speculation that EgTER1 might represent a candidate for enoyl-acyl-ACP reductase activity, as-yet unidentified, in the chloroplastic FAS II pathway. Although Hoffmeister et al [13] failed to detect the NADH-dependent crotonyl-CoA reductase activity in chloroplasts purified from *Euglena* cells, this is not decisive evidence that EgTER1 is a non-chloroplastic enzyme. Further analysis will be required to clarifying whether this enzyme can function in the FAS II system for chloroplast development.

Taken together, these findings indicate that EgTER1 constitutes a major isoform of crotonyl-CoA reductases, albeit is dispensable for wax ester production under anaerobic conditions. How *Euglena* produces wax esters without TER activity is currently unknown. Further detailed biochemical and metabolomic analyses combined with reverse genetics will be required to answer this question in future studies.

## Supporting information

S1 Fig Effect of EgTER1-knockdown on cell growth and chlororphyll content in autotrophically grown cells. (a) RT-PCR for verification of *EgTER1* gene knockdown. RT-PCR was carried out using total RNA from *Euglena* cells in which dsRNA was introduced. Mock cells electroporated without dsRNA. (b) Green color-less phenotype of KD-*ter1* cells. The picture shows a representative culture after 15 days growth in the autotrophic CM medium. (c) Growth curve of mock control (blue) and KD-*ter1* (orange) cells. The cultures were incubated in the autotrophic CM medium. An asterisk denotes statistically significant differences (*P< 0.05) compared with the mock control. Values represent the means ± SD of three independent experiments. (d) Total chlorophyll content. An asterisk denotes statistically significant differences (*P< 0.05) compared with the mock control. Values represent the means ± SD of three independent experiments.
(PDF)

S2 Fig Confirmation of the bleaching phenotype of *EgTER1*-knockdown cells by using other regions of *EgTER1* sequence as triggers for RNAi. (a) *EgTER* mRNA organization and positions of primers used for the gene knockdown. Each arrowhead pair with different colors indicates the regions where individual dsRNAs were generated. (b) RT-PCR for verification of *EgTER1* gene knockdown. (c) Green color-less phenotype of KD-*ter1* cells. The picture shows a representative culture after 7 days growth in the heterotrophic KH medium. (d) Total chlorophyll content. An asterisk denotes statistically significant differences (*P< 0.05, **P< 0.01) compared with the mock control. Values represent the means ± SD of three independent experiments. (e) Myristyl myristate content. *Euglena* cells grown to stationary phase were anaerobically treated for 24 h and collected for wax ester measurement. An asterisk denotes statistically significant differences (*P< 0.05) compared with the mock control. Values represent the means ± SD of three independent experiments.
(PDF)

S3 Fig Comparison of fatty acids profile in mock and EgTER1-knockdown cells. *Euglena* cells grown to stationary phase were anaerobically treated for 24 h and then collected for fatty acids measurement as described in the Material and Method section.
(PDF)

S4 Fig Putative transmembrane domains predicted by the TMHMM program (http://www.cbs.dtu.dk/services/TMHMM/) and putative motif domains indicated by the Pfam database (http://pfam.xfam.org/). The top picture shows the predicted known motifs in each EgTER. The striped profile in the bottom picture shows the probability for TM helix.
(PDF)

S1 Table Primer list. (PDF)

S2 Table Prediction of subcellular localization of *Euglena* TER isoforms using TargetP. (PDF)
