# Supplementary figures and images for: A major isoform of mitochondrial trans-2-enoyl-CoA reductase is dispensable for wax ester production in Euglena gracilis under anaerobic conditions
Source: PLoS One. 2019 Jan 16;14(1):e0210755. doi: 10.1371/journal.pone.0210755 (PMC6334954; doi:10.1371/journal.pone.0210755)

(a)

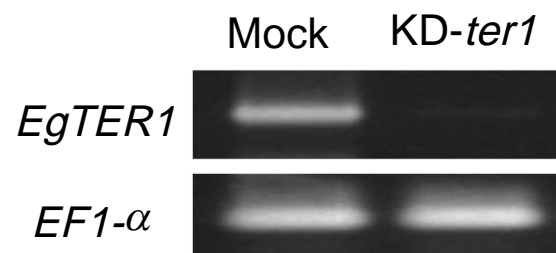

(b)

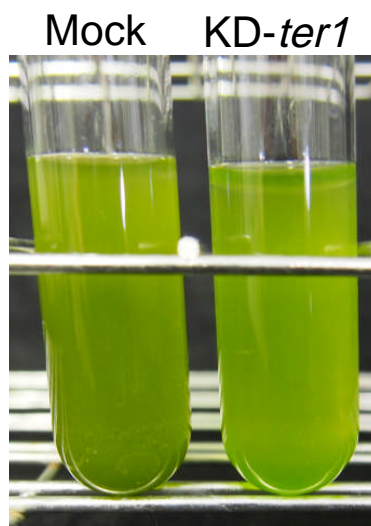

(c)

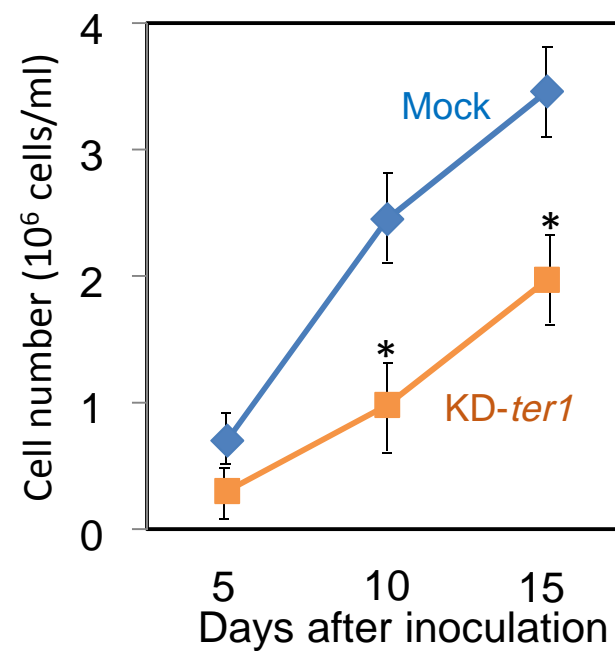

(d)

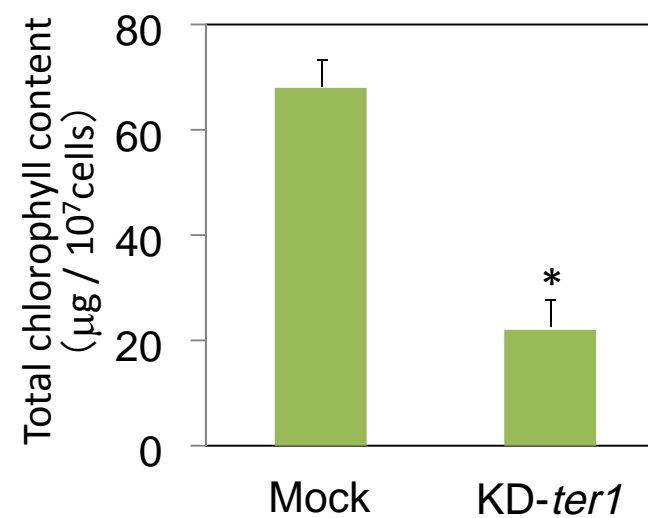

Supplement: S1 Fig — (a) RT-PCR for verification of EgTER1 gene knockdown. RT-PCR was carried out using total RNA from Euglena cells in which dsRNA was introduced. Mock cells electroporated without dsRNA. (b) Green color-less phenotype of KD-ter1 cells. The picture shows a representative culture after 15 days growth in the autotrophic CM medium. (c) Growth curve of mock control (blue) and KD-ter1 (orange) cells. The cultures were incubated in the autotrophic CM medium. An asterisk denotes statistically significant differences (*P< 0.05) compared with the mock control. Values represent the means ± SD of three independent experiments. (d) Total chlorophyll content. An asterisk denotes statistically significant differences (*P< 0.05) compared with the mock control. Values represent the means ± SD of three independent experiments. (PDF) [file pone.0210755.s001.pdf]

(a) TER1 (Comp34527)

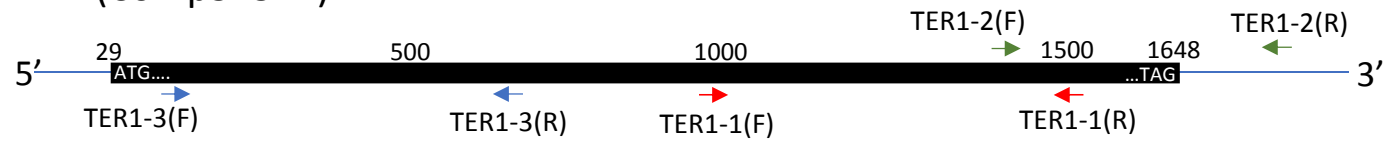

(b)

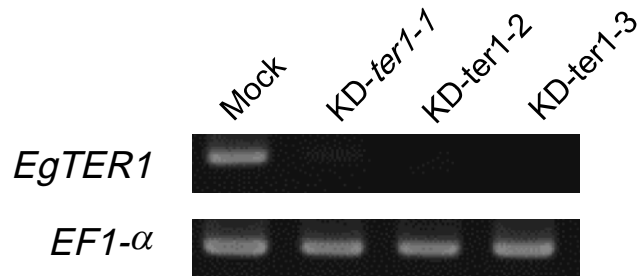

(c)

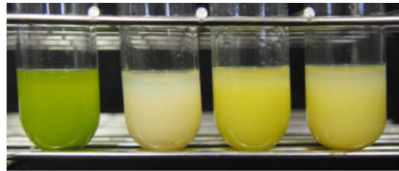

(d)

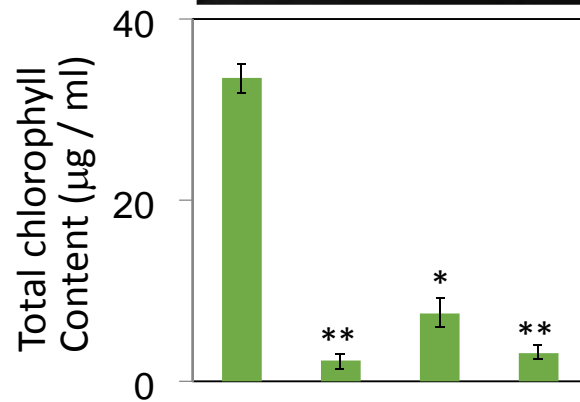

(e)

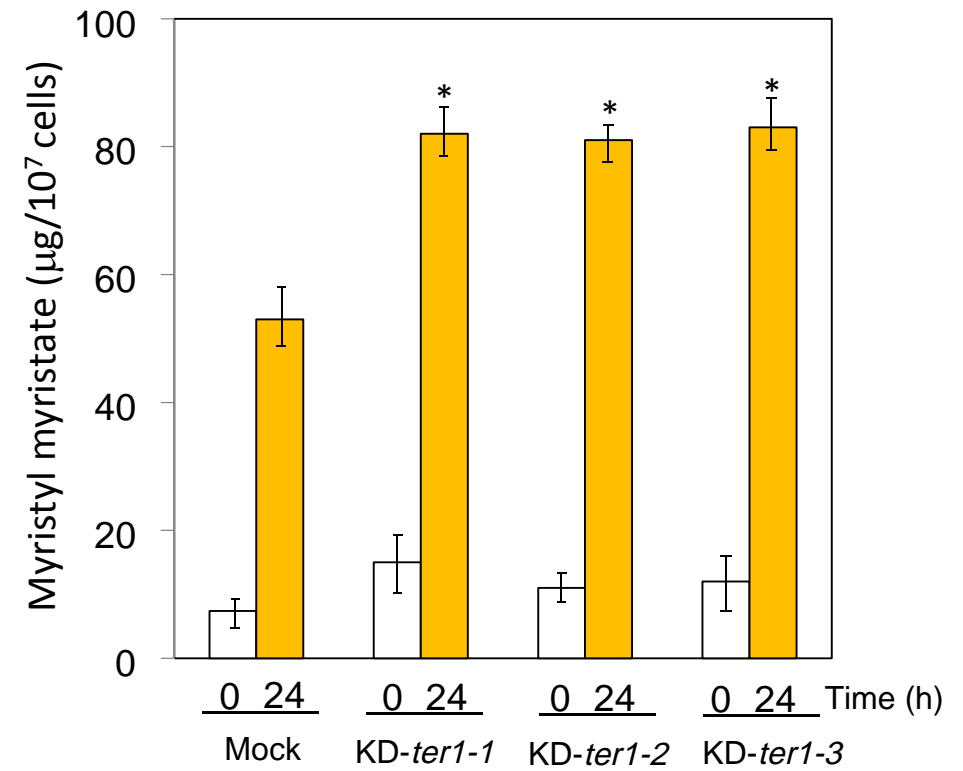

Supplement: S2 Fig — (a) EgTER mRNA organization and positions of primers used for the gene knockdown. Each arrowhead pair with different colors indicates the regions where individual dsRNAs were generated. (b) RT-PCR for verification of EgTER1 gene knockdown. (c) Green color-less phenotype of KD-ter1 cells. The picture shows a representative culture after 7 days growth in the heterotrophic KH medium. (d) Total chlorophyll content. An asterisk denotes statistically significant differences (*P< 0.05, **P< 0.01) compared with the mock control. Values represent the means ± SD of three independent experiments. (e) Myristyl myristate content. Euglena cells grown to stationary phase were anaerobically treated for 24 h and collected for wax ester measurement. An asterisk denotes statistically significant differences (*P< 0.05) compared with the mock control. Values represent the means ± SD of three independent experiments. (PDF) [file pone.0210755.s002.pdf]

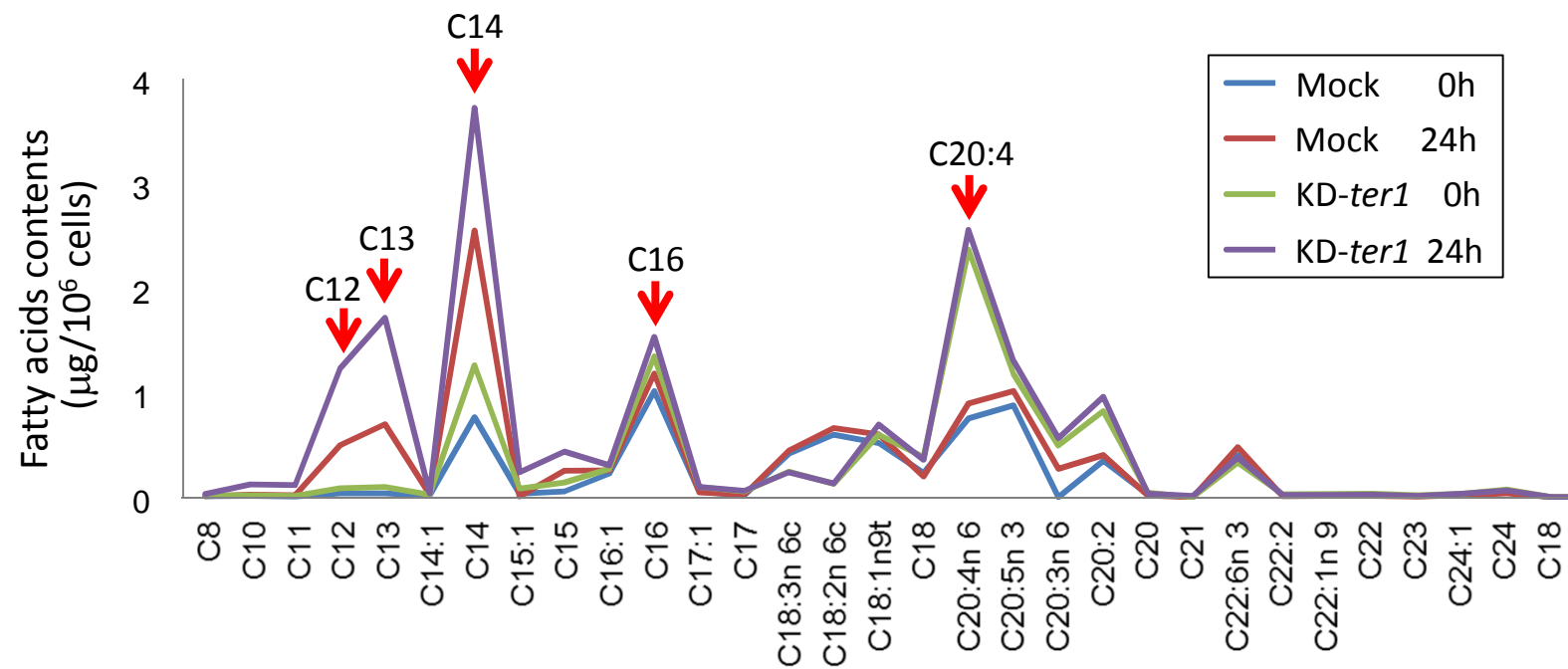

S3 Figure

Supplement: S3 Fig — Euglena cells grown to stationary phase were anaerobically treated for 24 h and then collected for fatty acids measurement as described in the Material and Method section. (PDF) [file pone.0210755.s003.pdf]
