# Supplementary material for: A major isoform of mitochondrial trans-2-enoyl-CoA reductase is dispensable for wax ester production in Euglena gracilis under anaerobic conditions
Source: PLoS One. 2019 Jan 16;14(1):e0210755. doi: 10.1371/journal.pone.0210755 (PMC6334954; doi:10.1371/journal.pone.0210755)
Supplement: S4 Fig — The top picture shows the predicted known motifs in each EgTER. The striped profile in the bottom picture shows the probability for TM helix. (PDF) [file pone.0210755.s004.pdf]

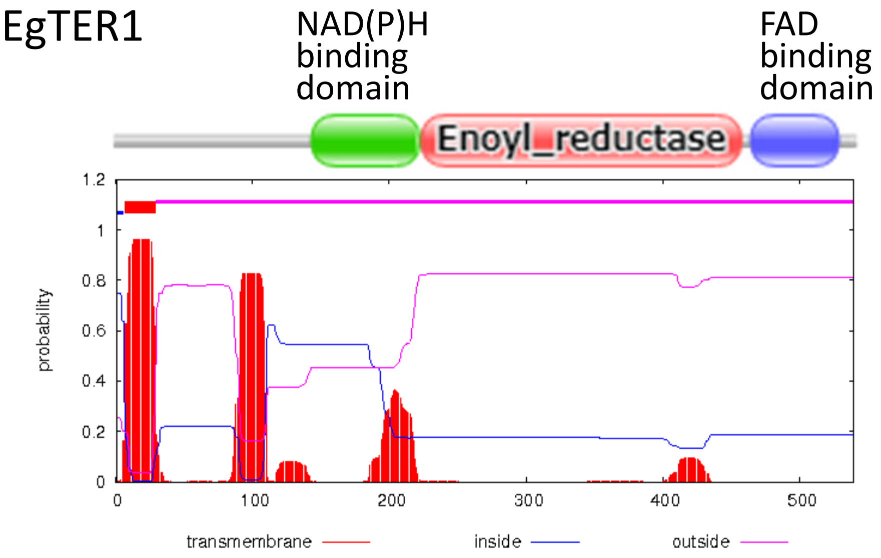
EgTER1
NAD(P)H binding domain
Enoyl_reductase
FAD binding domain
probability
1.2
1
0.8
0.6
0.4
0.2
0
0
100
200
300
400
500
transmembrane
inside
outside

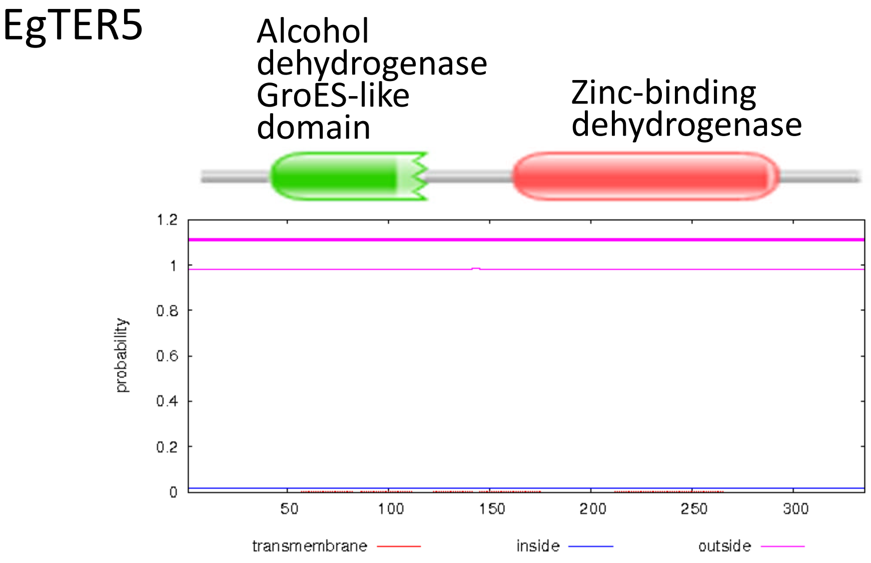
EgTER5
Alcohol dehydrogenase GroES-like domain
Zinc-binding dehydrogenase
probability
1.2
1
0.8
0.6
0.4
0.2
0
50
100
150
200
250
300
transmembrane
inside
outside

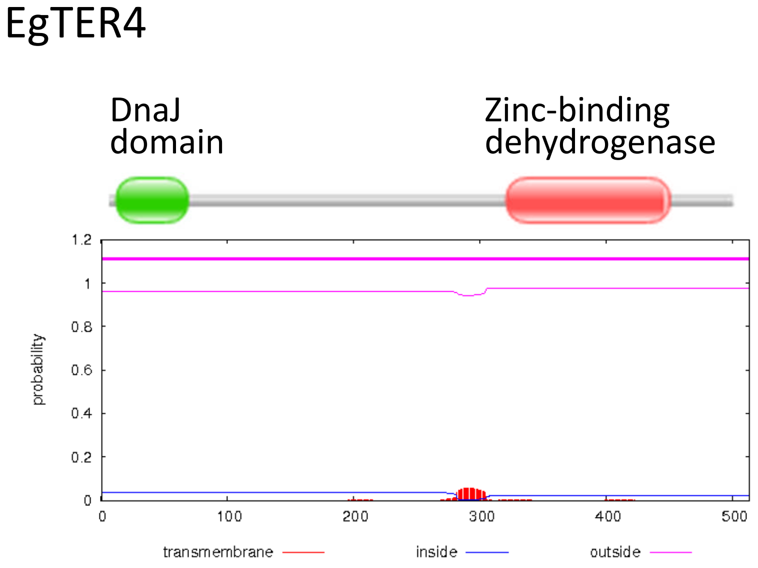
EgTER4
DnaJ domain
Zinc-binding dehydrogenase
probability
1.2
1
0.8
0.6
0.4
0.2
0
0
100
200
300
400
500
transmembrane
inside
outside

S4 Figure
