# Supplementary material for: A major isoform of mitochondrial trans-2-enoyl-CoA reductase is dispensable for wax ester production in Euglena gracilis under anaerobic conditions
Source: PLoS One. 2019 Jan 16;14(1):e0210755. doi: 10.1371/journal.pone.0210755 (PMC6334954; doi:10.1371/journal.pone.0210755)
Supplement: S1 Table — (PDF) [file pone.0210755.s005.pdf]

S1 Table　Primer list

Primer name	Target cDNA	oligonucleotide
RNAi experimets		
TER1-RNAi Fw	EgTER1	TAATACGACTCACTATAGGGGGTATTGTTGGCGACTGG
TER1-RNAi Rv	EgTER1	TAATACGACTCACTATAGGGGAACGCCACGGTGTTG
TER1-2-RNAi Fw	EgTER1	TAATACGACTCACTATAGGGAGGAGAAGGGCACCCACGAG
TER1-2-RNAi Rv	EgTER1	TAATACGACTCACTATAGGGGGCACAATTATGGCCCGTGT
TER1-3-RNAi Fw	EgTER1	TAATACGACTCACTATAGGGGCCTGTGCGTGGCAACGGTA
TER1-3-RNAi Rv	EgTER1	TAATACGACTCACTATAGGGAGAGCCCGTAGCCGGTACTG
TER2-RNAi Fw	EgTER2	TAATACGACTCACTATAGGGAAAGCCGCCCACCTCAAGGC
TER2-RNAi Rv	EgTER2	TAATACGACTCACTATAGGGACGGCGTTATGGGTGCCGTG
TER3-RNAi Fw	EgTER3	TAATACGACTCACTATAGGGGGGCAGCTACGCCACGCTGG
TER3-RNAi Rv	EgTER3	TAATACGACTCACTATAGGGGCAGCTTGGCGAGGTCGTGC
TER4-RNAi Fw	EgTER4	TAATACGACTCACTATAGGGTCACCTCCGACACCTTGATG
TER4-RNAi Rv	EgTER4	TAATACGACTCACTATAGGGCCTTCTTTGAGGGTCTCCAC
TER5-RNAi Fw	EgTER5	TAATACGACTCACTATAGGGGTTATTGGTGGTGAGGGCTG
TER5-RNAi Rv	EgTER5	TAATACGACTCACTATAGGGCTTGAAGATGAGCTGACCCC
RT-PCR		
TER1-RT Fw	EgTER1	AGACGGTGGCGTACTCC
TER1-RT Rv	EgTER1	CAATGCCGAACCCGAAC
TER2-RT Fw	EgTER2	TGCCAAAGCTTGGGGCATC
TER2-RT Rv	EgTER2	CCAGCTTGCCACGAAGCTC
TER3-RT Fw	EgTER3	ACCAAATCCTGAAGCTCTCG
TER3-RT Rv	EgTER3	CCATATGAAAACCGAGGGAC
TER4-RT Fw	EgTER4	ACACGCTGCAGCGGCAGATG
TER4-RT Rv	EgTER4	CTGACCACGGCCGGGGTGAG
TER5-RT Fw	EgTER5	ATCAACGTCATGCAGGGTGTC
TER5-RT Rv	EgTER5	GCTTCAGATGGAAGCCCACG
EF1α-F	EgEF1α	ACAGATTGGGAACGGGTACGC
EF1α-R	EgEF1α	CGCAGTTTCCCTTCACCATCG
Recombinant protein expression		
rTER1 Fw	EgTER1	GAATTCATGGCGATGTTCACCAC
rTER1 Rv	EgTER1	AAGCTTCGTCCAGCACCTACTGC
rTER2 Fw	EgTER2	CTCGAGCATGCTCATGCAG
rTER2 Rv	EgTER2	GGATCCTCAACTTGCAAACTCG
rTER3 Fw	EgTER3	GAGCTCATGGCCCTTGCCAAAGGCTG
rTER3 Rv	EgTER3	AAGCTTTAGGAGTGCGTCGGGTCAGC
rTER4 Fw	EgTER4	GAGCTCATGACCACGGGTCATCTCGAG
rTER4 Rv	EgTER4	AAGCTTCATGCGATTTTAACGTTCGG
rTER5 Fw	EgTER5	GAGCTCCTGGCGCTGCACCTCTACCAC
rTER5 Rv	EgTER5	AAGCTTCAGCTGGAAGAGATTCTCCCACATC
