# Supplementary material for: A major isoform of mitochondrial trans-2-enoyl-CoA reductase is dispensable for wax ester production in Euglena gracilis under anaerobic conditions
Source: PLoS One. 2019 Jan 16;14(1):e0210755. doi: 10.1371/journal.pone.0210755 (PMC6334954; doi:10.1371/journal.pone.0210755)
Supplement: S2 Table — (PDF) [file pone.0210755.s006.pdf]

S2 Table Prediction of subcellular localization of *Euglena* TER isoforms using TargetP

Name	Length	cTP	mTP	SP	other	Loc	RC
EgTER1	539	0.508	0.006	0.764	0.014	S	4
EgTER2	248	0.053	0.205	0.116	0.417	_	4
EgTER3	290	0.038	0.681	0.025	0.39	M	4
EgTER4	513	0.07	0.432	0.065	0.698	_	4
EgTER5	335	0.047	0.388	0.048	0.38	M	5
